# Generalized lymph node immunoglobulin G amyloidoma

**DOI:** 10.1007/s00277-012-1418-1

**Published:** 2012-02-09

**Authors:** Tao Chan, Henry K. F. Mak, Yok-Lam Kwong

**Affiliations:** 1 Department of Diagnostic Radiology, Queen Mary Hospital, Hong Kong, China; 2 Department of Medicine, Professorial Block, Queen Mary Hospital, Pokfulam Road, Hong Kong, China

**Fig. 1:**
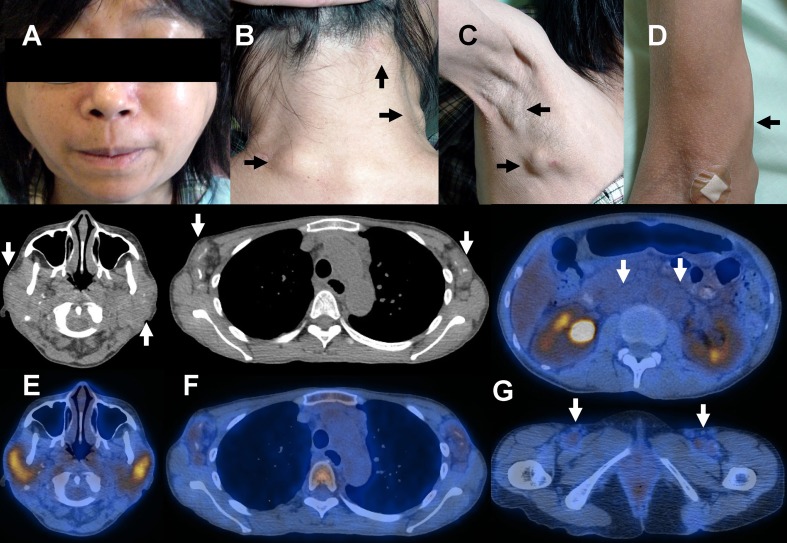
Patient with marked sicca symptoms. Bilateral parotid enlargement was found (**a**). There was generalized lymphadenopathy affecting the head and neck (**b**, *arrows*), axillae (**c**, *arrows*), epitrochlear (**d**, *arrow*), and groin regions. FDG PET/CT showed calcified parotid glands (**e**, *arrows*) with mild FDG uptake. There was generalized lymphadenopathy showing fine specks of calcification (**f**, *arrows*, axillae), with minimal FDG uptake. There was also para-aortic and groin lymphadenoapthy (**g**, *arrows*)

Dear Editor,

A 29-year-old woman presented with proteinuria. Renal biopsy showed features consistent with lupus nephritis, although she had otherwise no symptoms of systemic lupus erythematosus. Treatment with cyclophosphamide and corticosteroid led to remission of the proteinuria. However, the globulin level gradually increased, reaching 76 g/L after 11 years. Generalized lymphadenopathy was also found. Biopsy of a cervical lymph node showed amorphous materials that were salmon pink on Congo red staining, with apple green birefringence under polarized microscopy. Serum immunoelectrophoresis showed an immunoglobulin G lambda paraprotein. Treatment with thalidomide was ineffective. On referral 3 years later, the patient had marked sicca symptoms. Bilateral parotid enlargement was found (Fig. 1a). There was generalized lymphadenopathy affecting the head and neck (arrows, Fig. 1b), axillae (arrows, Fig. 1c), epitrochlear (arrow, Fig. 1d), and groin regions. ^18^F-fluorodeoxyglucose (FDG) positron emission tomography computed tomography (PET/CT) showed calcified parotid glands (arrows, Fig. 1e) with mild FDG uptake. There was generalized lymphadenopathy showing fine specks of calcification (arrows, axillae, Fig. 1f), with minimal FDG uptake. The para-aortic and groin lymphadenoapthy (arrows, Fig. 1g) showed similar features. Marrow examination showed 14% monotypic lambda expressing plasma cells. The overall features were consistent with generalized lymph node amyloidoma due to plasma cell dyscrasia, associated with an underlying Sjögren syndrome.

Amyloidoma refers to the unusual local depositions of amyloid, which present as apparent tumor masses. There is a predilection for the upper respiratory tract, central nervous system, long bones, and the spine. Generalized lymph node amyloidoma is highly unusual, due almost invariably to immunoglobulin M paraproteinemia, arising from an underlying lymphoproliferative disease [1, 2]. The unique features in this case include immunoglobulin G paraproteinemia due to a plasma cell dyscrasia, association with Sjögren syndrome, and massive lymph node involvement. On PET/CT, lymph node amyloidoma usually shows dystrophic calcification with minimal to no FDG uptake. This feature distinguishes nodal amyloidoma from nodal tuberculosis, sarcoidosis, or metastatic cancers, where calcified lymph nodes are hypermetabolic [2].
